# Histamine Released From Skin-Infiltrating Basophils but Not Mast Cells Is Crucial for Acquired Tick Resistance in Mice

**DOI:** 10.3389/fimmu.2018.01540

**Published:** 2018-07-03

**Authors:** Yuya Tabakawa, Takuya Ohta, Soichiro Yoshikawa, Elisabeth J. Robinson, Kayoko Yamaji, Kenji Ishiwata, Yohei Kawano, Kensuke Miyake, Yoshinori Yamanishi, Hiroshi Ohtsu, Takahiro Adachi, Naohiro Watanabe, Hirotaka Kanuka, Hajime Karasuyama

**Affiliations:** ^1^Department of Immune Regulation, Graduate School of Medical and Dental Sciences, Tokyo Medical and Dental University, Tokyo, Japan; ^2^Faculty of Medicine, Imperial College London, London, United Kingdom; ^3^Department of Tropical Medicine, The Jikei University School of Medicine, Tokyo, Japan; ^4^Tekiju Rehabilitation Hospital, Kobe, Japan; ^5^Department of Immunology, Medical Research Institute, Tokyo Medical and Dental University, Tokyo, Japan

**Keywords:** basophil, mast cell, histamine, tick resistance, epidermal hyperplasia

## Abstract

Ticks are blood-feeding arthropods that can transmit pathogens to humans and animals, leading to serious infectious diseases such as Lyme disease. After single or multiple tick infestation, some animal species develop resistance to tick feeding, leading to reduced risk of pathogen transmission. In mice infested with larval *Haemaphysalis longicornis* ticks, both mast cells and basophils reportedly play key roles in the manifestation of acquired tick resistance (ATR), but it remains ill-defined how they contribute to it. Here, we investigated their products responsible for ATR. Treatment of mice with antihistamine abolished the ATR while histamine or histamine H1 receptor agonist reduced tick-feeding even in the first infestation. In accordance with these, mice deficient for histamine production showed little or no ATR, indicating the crucial role for histamine in the expression of ATR. Adoptive transfer of mast cells and basophils derived from histamine-sufficient or deficient mice to recipient mice lacking mast cells and basophils, respectively, revealed that histamine produced by basophils but not mast cells is essential for the manifestation of ATR, in contrast to the case of local and systemic anaphylaxis where mast cell-derived histamine is the major player. During the second but not first tick infestation, basophils accumulated and made a cluster, surrounding a tick mouthpart, in the epidermis whereas mast cells were scattered and localized mainly in the dermis, more distantly from a tick mouthpart. This appears to explain why basophil-derived histamine is much more effective than mast cell-derived one. Histamine-sufficient, but not -deficient mice showed the thickened epidermis at the second tick-feeding site. Taken together, histamine released from skin-infiltrating basophils rather than skin-resident mast cells plays a crucial role in the manifestation of ATR, perhaps through promotion of epidermal hyperplasia that may inhibit tick feeding.

## Introduction

Ticks are blood-feeding ectoparasites of medical and veterinary public health importance (1). They are second to mosquitos as vectors of pathogens that cause infectious diseases, including Lyme disease, in humans (2, 3). Ticks insert their mouthparts through the host skin to feed blood meal, and hard ticks (Ixodidae), in particular, require several days to weeks to replete with blood meal and drop off from the host (3). During this feeding process, tick saliva containing various substances is injected into the host to facilitate their blood feeding (4, 5). Pathogens can be transmitted from infected ticks to the host during salivation, leading to serious infectious diseases in the host.

Some animals, including cattle, rabbits, guinea pigs, and mice have been reported to develop resistance to tick feeding once they have experienced tick infestation (6), depending on the combination of tick species and animal species/strains. Acquired tick resistance (ATR) is manifested by reduced numbers and body weights of engorged ticks or tick death in subsequent infestations. Of note, ATR can reduce the risk of pathogen transmission from infected ticks to the host (5). Therefore, it is important to elucidate cellular and molecular mechanisms underlying ATR, facilitating the development of efficient vaccines against tick infestation. ATR is not limited to the site of previous tick feeding, suggesting the involvement of systemic responses rather than a localized alteration of the skin. At the cellular level, basophils and mast cells have been demonstrated to play a crucial role in the manifestation of ATR (7–9). Tick feeding sites of previously infested guinea pigs are characterized by accumulation of basophils and eosinophils (10). Basophils represent up to 70% of cellular infiltrates, and their ablation using a basophil-depleting antiserum resulted in abolished ATR (7), indicating the importance of basophils in the manifestation of ATR. In mice, it was first reported that mast cells instead of basophils are essential for ATR (8), based on the observations that basophils were hardly detected at the second tick-feeding site in mice unlike in guinea pigs, and that mast cell-deficient mice failed to manifest ATR. We later identified the accumulation of basophils at the second tick-feeding site in mice (9) by taking advantage of a newly generated mAb specific to mMCP-8, a serine protease that is selectively expressed by murine basophils (11). Even though basophils accounted for less than 5% of cellular infiltrates unlike in guinea pigs, the depletion of basophils before the second tick infestation almost completely abolished ATR (9). Thus, basophils are key players in the manifestation of ATR in both guinea pigs and mice. Importantly, we confirmed the essential role of mast cells in ATR in the case of C57BL/6 mice infested with *Haemaphysalis longicornis* (*H. longicornis*) ticks (9). Therefore, both basophils and mast cells contribute to the manifestation of ATR in this setting while the contribution of mast cells in guinea pigs remains to be determined.

In the present study, we sought to elucidate basophils/mast cell-derived effector molecules responsible for the manifestation of ATR. Biologically active molecules, including histamine and proteases stored in their secretory granules, are implicated in the expression of ATR. In cattle, the tick resistance is reportedly correlated with hypersensitivity to tick antigens and with the amounts of histamine at the tick feeding site (12). Higher tick numbers were observed in cattle treated with an antihistamine (13) while administration of histamine into the cattle skin resulted in increased tick detachment (14). Similar observations were reported in guinea pigs as well (15), suggesting the possible involvement of histamine to the manifestation of ATR. However, the cellular source of histamine responsible for ATR and the mechanism underlying histamine-elicited ATR remain poorly understood. To address these issues in the present study, we used the mouse model of infestation with *H. longicornis* tick larvae, demonstrating that histamine released from basophils rather than mast cells plays a key role in ATR, perhaps through promotion of epidermal hyperplasia at the tick feeding site.

## Materials and Methods

### Mice

C57BL/6 mice were purchased from SLC, Japan. Histidine decarboxylase (HDC)-deficient, histamine H1 receptor (H1R)-deficient, mast cell-deficient (*Kit^W-sh/W-sh^*), and inducible basophil-deficient (*Mcpt8^DTR^*) mice on the C57BL/6 background were described previously (9, 16, 17, 18). *Kit^W-sh/W-sh^* mice carrying green fluorescent protein (GFP)-expressing basophils (*Kit^W-sh/W-sh^Mcpt8*^GFP^) were generated by crossbreeding of *Kit^W-sh/W-sh^* mice with *Mcpt8*^GFP^ mice (19). CAG-tdTomato transgenic mice were obtained by crossbreeding CAG-Cre transgenic mice with tdTomato-floxed mice (Adachi T. et al., unpublished). Mice were maintained under specific pathogen-free conditions in our animal facilities. All animal studies were approved by institutional Animal Care and Use Committee of Tokyo Medical and Dental University (Permission number #0170087A).

### Reagents

Biotin-conjugated CD49b (DX5), Allophycocyanin-conjugated CD200R3 (Ba13), anti-CD49b (HMα2), anti-CD63 (NVG-2), rat IgG2a isotype control antibody (RTK2758), Fluoresceinisothiocyanate-conjugated anti-CD49b (HMα2), Pacific Blue™-conjugated anti-CD117 (2B8), PE/Cy7-conjugated anti-CD45 (30-F11), and recombinant murine IL-3 and SCF were purchased from BioLegend. Following reagents were from Sigma-Aldrich: diphtheria toxin (DT), pyrilamine maleate salt (H1R antagonist), and cimetidine (histamine H2 receptor antagonist). 2-pyridylethylamine dihydrochloride (H1R agonist), dimaprit dihydrochloride (histamine H2 receptor agonist), (R)-(-)-α-methylhistamine dihydrobromide (histamine H3 receptor agonist), and VUF8430 dihydrobromide (histamine H4 receptor agonist) were obtained from Tocris Bioscience. 2,4,6-trinitrophenol (TNP)-specific immunoglobulin E (IgE) (TNP-IgE, IGEL-b4) and anti-CD16/32 (2.4G2) mAb were prepared in our laboratory from ascites generated in mice receiving mAb-producing hybridomas.

### Preparation of Bone Marrow-Derived Basophils (BMBAs) and Mast Cells

Cultured BMBAs and bone marrow-derived mast cells (BMMCs) were prepared from WT or (HDC)-deficient mice as described previously (11) with some modification. To generate and isolate BMBAs, bone marrow cells were cultured in the presence of 100 pg/ml IL-3 for 6 days, followed by enrichment of CD49b^+^ cells by using biotinylated anti-CD49b antibody and IMag system (BD Biosciences). To generate BMMCs, bone marrow cells were cultured in the presence of 3 ng/ml IL-3 and 15 ng/ml SCF for 4 weeks.

### Histamine Release Assay

Bone marrow-derived basophils or BMMCs (5 × 10^5^ cells) were sensitized with TNP-specific IgE and then stimulated for 2 h with 300 ng/ml TNP-conjugated ovalbumin (TNP-OVA) or control OVA in Tyrode’s buffer. Supernatants were then collected for examination of histamine release assay with histamine ELISA kit (Bertin Pharma).

### Tick Infestation

Mice were infested once or twice with 40 larvae of *H. longicornis* ticks at each time, as reported previously (9, 19). To prevent the effect of mouse grooming on tick attachment and blood feeding, larvae were placed into a short piece of acrylic pipe that was attached to the shaved skin, and the open end of the pipe was covered with nylon gauze. To assess the acquisition of tick resistance, mice were infested with tick larvae at two different locations. The first infestation was on the left flank. Most of the ticks became engorged and detached from hosts within 6 days. The second infestation was conducted on the right flank, starting 14 days after the initiation of the first infestation. For evaluation of acquired resistance to tick feeding, we summed up the body weight of all engorged ticks in each mouse as our measure of tick feeding and calculated “relative tick repletion.” Relative tick repletion (%) = 100 × the sum of the body weights of all engorged ticks in the test experiment/the sum of the body weights of all engorged ticks in the reference experiment. When the tick repletion in the first infestation was defined as 100%, which in the second infestation was typically 40–60% in C57BL/6 mice. Because the body weight of unengorged ticks is negligible compared to that of engorged ones, the relative tick repletion in the second infestation compared to the first one actually represents the ratio of the total weight of all ticks in the second infestation to that in the first infestation.

### Treatment of Mice With Histamine, Histamine Receptor Agonists, or Antagonists

C57BL/6 mice were treated with intradermal administration of 2 µmol histamine, histamine receptor agonists, or control PBS beneath the tick-infested site once a day for 7 days, starting 1 day before the tick infestation. For blocking the effect of histamine, mice were treated with intravenous administration of 10 µmol histamine antagonists or control PBS once a day for 7 days, starting 1 day before the first or second infestation.

### Flow Cytometry

Single-cell suspensions were prepared from tick-feeding sites of the skin, as reported previously (19). After incubation with anti-CD16/32 mAb and normal rat serum to prevent the non-specific binding of irrelevant Abs, cells were stained with indicated combination of Abs, and analyzed with FACSCanto™ II (BD Biosciences) and FlowJo (TreeStar). Each cell lineage was defined as follows: basophils (CD49b^+^c-kit^−^CD200R3^+^), skin-resident mast cells (CD49b^+^c-kit^+^CD200R3^+^).

### Adoptive Transfer of Mast Cells and Basophils

Adoptive transfer of BMMCs was performed as reported previously (9). A total of 10^6^ BMMCs prepared from WT or HDC-deficient mice was intradermally administered into the right flank of mast cell-deficient *Kit^W-sh/W-sh^* mice. Four weeks later, the mice were infested with tick larvae, first on the left flank, and then, 2 weeks later, re-infected with larvae on the BMMC-injected site of the right flank. For confocal fluorescence microscopic examination, BMMCs were generated from CAG-tdTomato transgenic mice and intradermally administered into the right flank of mast cell-deficient *Kit^W-sh/W-sh^Mcpt8*^GFP^ mice in that only basophils express GFP. For adoptive transfer of basophils, basophils were isolated as follows. CD49b^+^ cells were enriched, by using IMag system (BD Biosciences), from splenic and bone marrow cells that were isolated from mice on day 13 of the first infestation (just before the second infestation), and CD49b^+^CD45^int^ cells were further sorted with FACSAria™ (BD) to obtain highly purified basophils (>95% CD200R3^+^c-kit^−^). *Mcpt8^DTR^* mice were first infested with tick larvae on the left flank, and then treated with intravenous administration of DT (750 ng/20 g body weight) 1 day before the second infestation to deplete basophils. Two hours before the second infestation, 10^5^ purified basophils from WT or HDC-deficient mice were intravenously administered into the DT-treated mice to reconstitute basophils.

### *In Vivo* Fluorescence Imaging

Mice were anesthetized with 1.5% isoflurane gas and covered with a heating blanket to keep their body temperature at 37°C. Intravital images of the flank skin were captured with an inverted laser scanning microscope (A1R+; Nikon) equipped with CFI Plan Apochromat λ 10× or 20× objective lens. NIS elements software was used for acquisition and analysis of images.

### Statistical Analysis

Comparisons between two parameters were analyzed unpaired Student’s *t*-test. When more groups were compared, one-way ANOVA was applied using the Graphpad Prism 7.03 software. A *p* value < 0.05 was considered statistically significant.

## Results

### Tick Resistance Is Abrogated in Mice by H1 Antihistamine and Can Be Generated by Histamine or H1R Agonist

**Figure 1:**
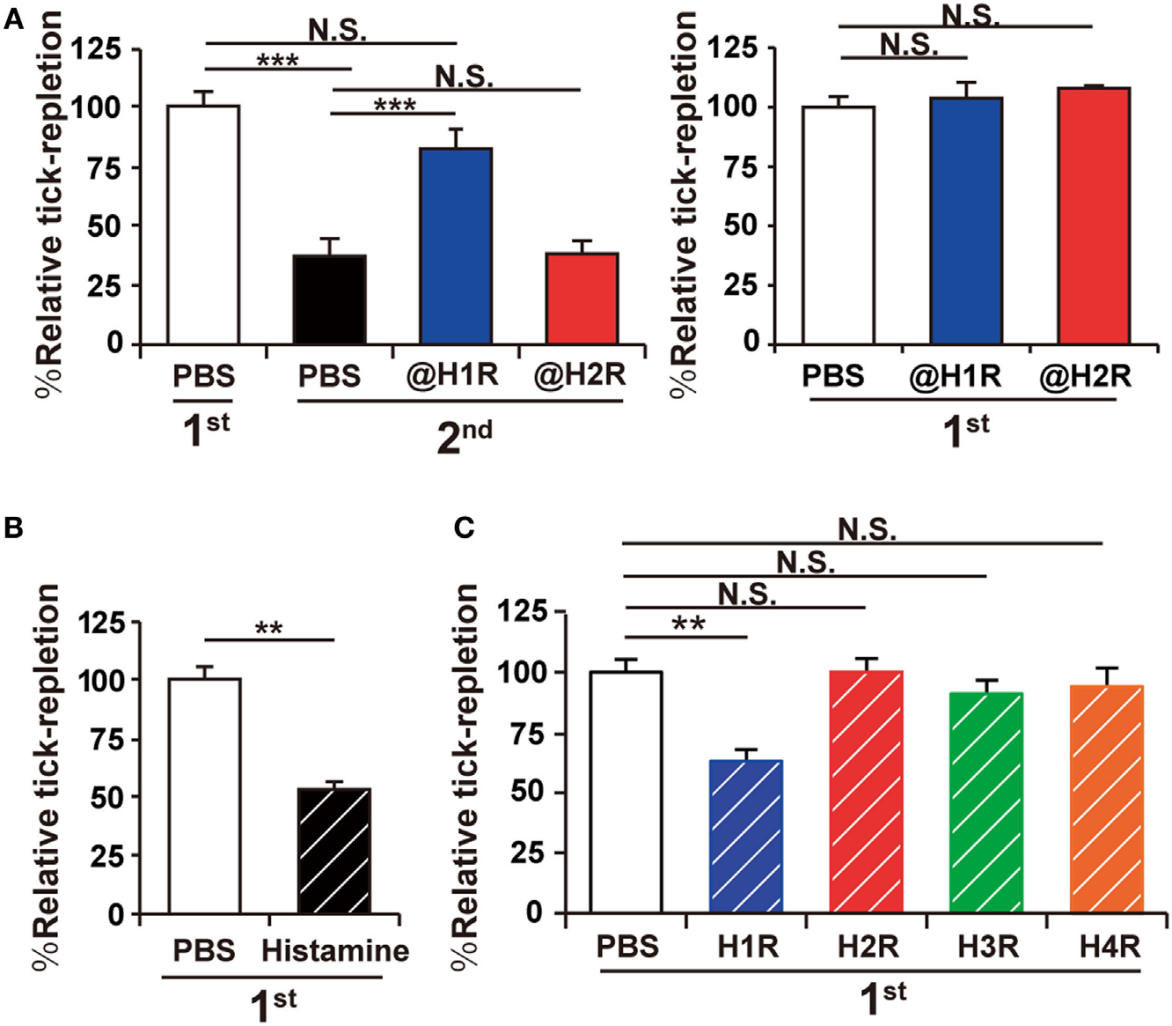
Acquired tick resistance is abrogated by a histamine H1 receptor antagonist. **(A)** Mice were infested with tick larvae once (right panel) or twice (left panel). They were treated with intravenous administration of histamine H1 or H2 receptor antagonist (@H1R or @H2R) or control PBS once a day for 7 days, starting 1 day before the first infestation (left panel) or the second infestation (right panel). The relative tick repletion in each experimental group is shown (mean ± SEM, *n* = 3 each), in that the value in the first infestation of mice treated with control PBS was defined as 100%. **(B,C)** Mice were infested once with tick larvae. Histamine **(B)**, histamine receptor (H1R, H2R, H3R or H4R) agonists **(C)**, or control PBS was injected intradermally beneath the tick-infested site once a day for 7 days, starting 1 day before the infestation. The relative tick repletion was calculated and shown as in **(A)** (mean ± SEM, *n* = 3–4). All the data shown are representative of three independent experiments. Statistics: *t-*test in **(B)** and one-way ANOVA in **(A)** and **(C)** N.S., not significant; **p < 0.01; ***p < 0.001.

Previous studies in cattle and guinea pigs suggested the contribution of histamine to ATR (13, 15), but it remains to be determined whether this can be applied to other animal species such as mice. To address this, C57BL/6 mice were infested with larval *H. longicornis* ticks once or twice in distant places of the skin at an interval of 14 days. The extent of tick feeding in the second infestation decreased to approximately half of that in the first one under our experimental conditions (Figure 1A, left panel), demonstrating the acquisition of tick resistance in previously infested mice. Treatment of mice with histamine H1 but not H2 receptor antagonist before and during the second infestation abolished this resistance (Figure 1A, left panel), while the same treatment in the first infestation showed no apparent effect on tick feeding (Figure 1A, right panel). These findings suggested the contribution of histamine to the manifestation of acquired, but not innate, resistance to tick feeding in mice. Consistent with this assumption, repeated intradermal injection of histamine beneath the tick-infested site before and during the first infestation reduced the tick feeding in the first infestation to approximately half of that in PBS-treated control mice (Figure 1B), as observed in the second infestation (Figure 1A, left panel). Repeated intradermal injection of H1R agonist showed the similar inhibitory effect on tick feeding during the first infestation whereas none of histamine H2, H3, and H4 agonists did so (Figure 1C). Taken together, these observations indicated the crucial role of the histamine–H1R axis in the manifestation of ATR in mice.

### HDC-Deficient Mice Show Little or no ATR

**Figure 2:**
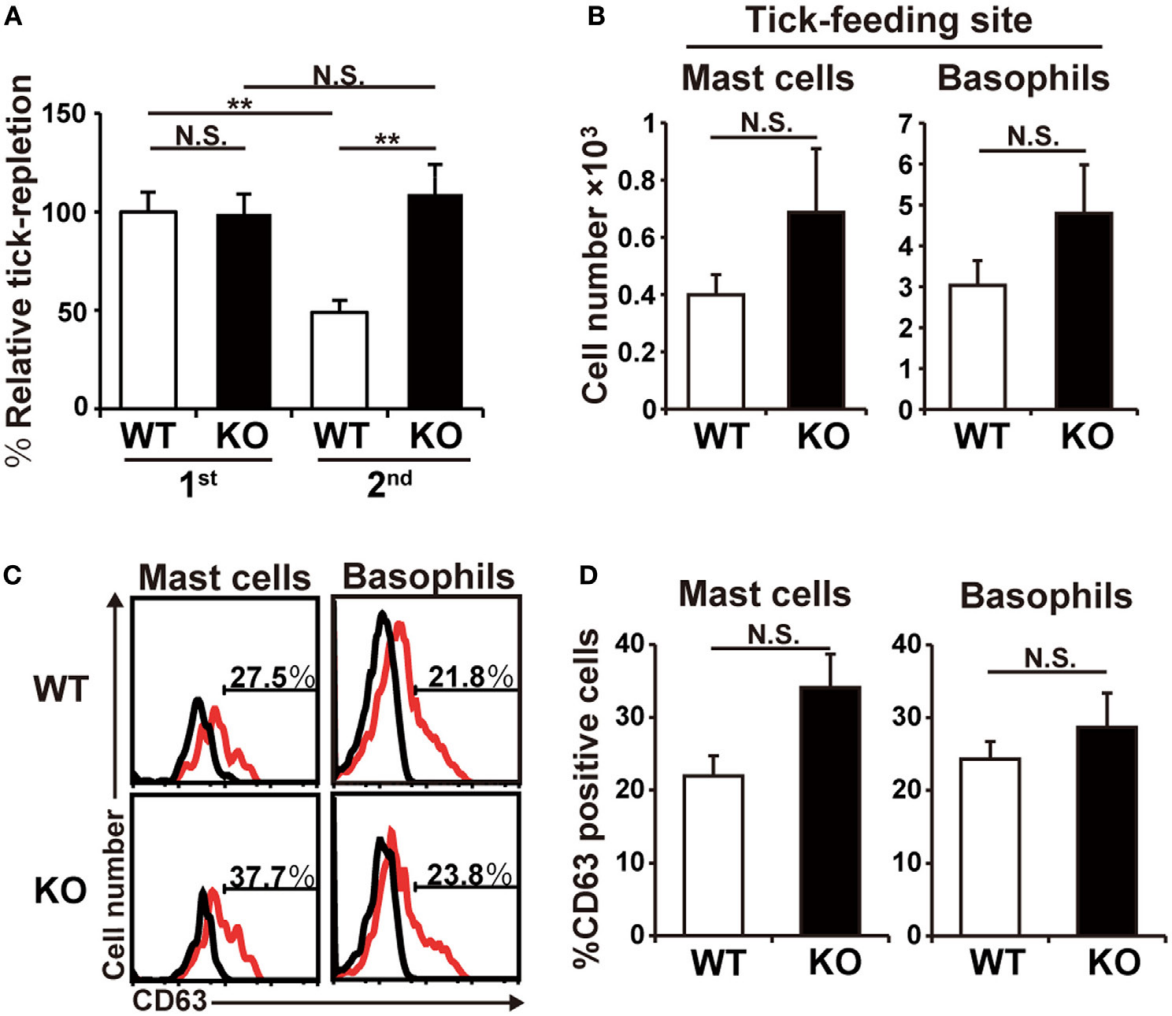
Histidine decarboxylase (HDC)-deficient mice show little or no acquired tick resistance. **(A)** WT and HDC-deficient (KO) mice were infested once or twice with tick larvae. The relative tick repletion was calculated and shown as in Figure 1A (mean ± SEM, *n* = 3–4). **(B–D)** WT and KO mice were infested twice with tick larvae. In **(B)**, the number of basophils and mast cells accumulating at the second tick-feeding site on day 2 of the second infestation is shown (mean ± SEM, *n* = 4 each). In **(C)**, surface expression of CD63 (red histograms) on basophils and mast cells isolated from the second tick-feeding site on day 2 of the second infestation is shown. Black histograms indicate control staining with an isotype-matched antibody. In **(D)**, the frequency (%) of CD63^+^ cells among basophils and mast cells examined in **(C)** is summarized (mean ± SEM, *n* = 3 each). All the data shown are representative of three independent experiments. N.S., not significant; **p < 0.01.

The observations shown in Figure 1 prompted us to examine ATR in HDC-deficient mice. HDC catalyzes biosynthesis of histamine from histidine and is a major generator of histamine in immune cells (20). In accordance with the experiments using antihistamine shown in Figure 1A, the deficiency of HDC showed no significant effect on the tick feeding in the first infestation, while it abolished ATR in the second infestation (Figure 2A). Previous studies demonstrated the key roles of both mast cells and basophils in the manifestation of ATR (13). The numbers of these cells detected at the second tick feeding site were comparable in wild-type (WT) and HDC-deficient mice (Figure 2B). The activation status of mast cells and basophils, assessed by the surface expression of CD63, was also comparable between these two mouse strains (Figures 2C,D; Figure S1 in Supplementary Material). Therefore, the loss of ATR in HDC-deficient mice was likely attributed to the failure in histamine synthesis rather than impaired accumulation or activation of mast cells and basophils in the absence of HDC.

### Histamine Derived From Basophils But not Mast Cells Is Crucial for the Manifestation of ATR

**Figure 3:**
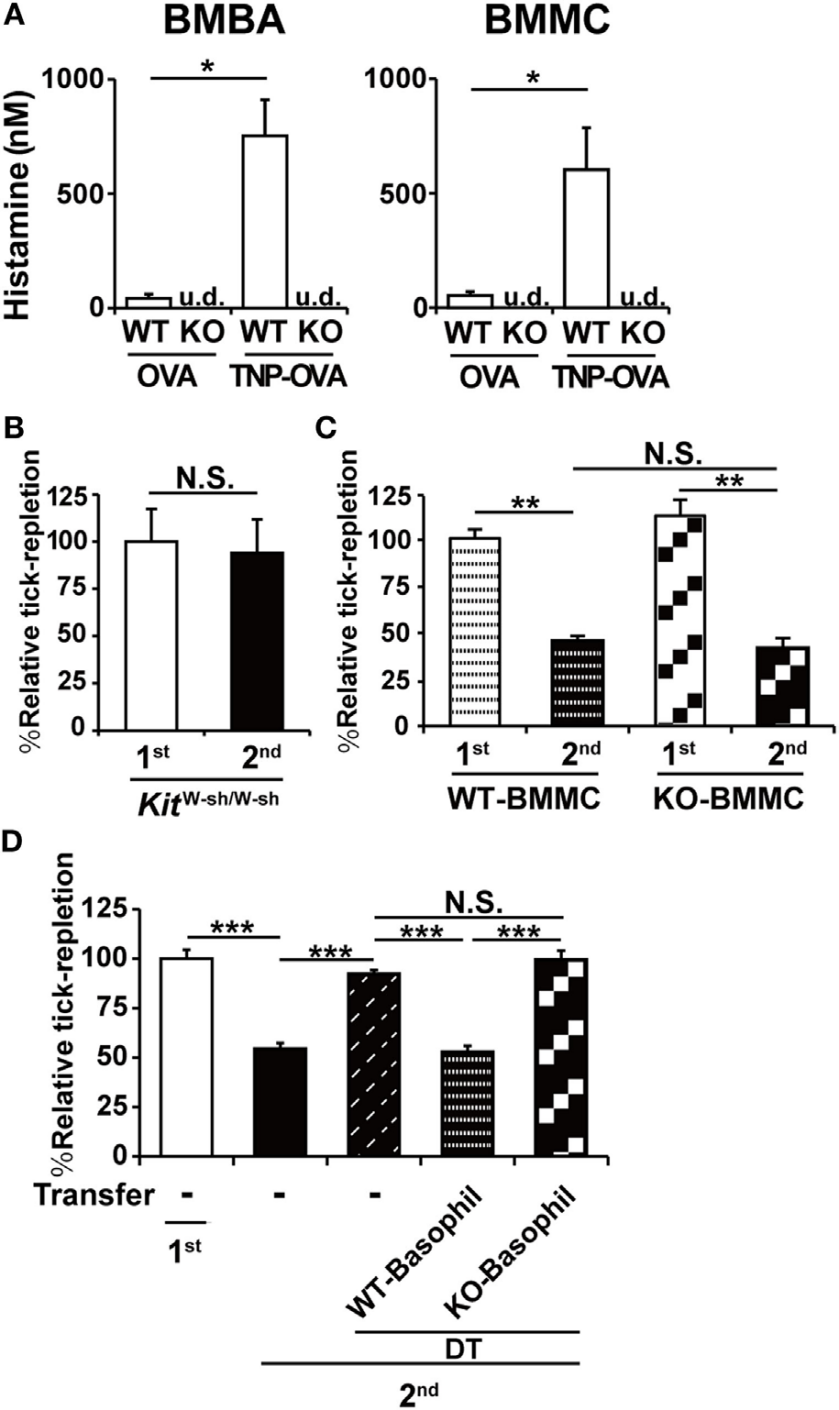
Histamine derived from basophils but not mast cells is crucial for the manifestation of acquired tick resistance. **(A)** Bone marrow-derived basophils and bone marrow-derived mast cell (BMMCs) generated from WT or histidine decarboxylase (HDC) KO mice were sensitized with anti-trinitrophenol (TNP)-immunoglobulin E and then stimulated with TNP-OVA or control OVA. The concentration of histamine released into culture medium is shown (mean ± SEM, *n* = 3). u.d., undetected **(B)** Mast cell-deficient *Kit^W-sh/W-sh^* mice were infested once or twice with ticks. The relative tick repletion is shown (mean ± SEM, *n* = 3 each), in that the value in the first infestation was defined as 100%. **(C)** BMMCs prepared from WT or HDC KO mice were intradermally administered into the right flank of *Kit^W-sh/W-sh^* mice. One group (first) of the recipient mice were infested once with tick larvae on the BMMC-injected site 6 weeks after the transplantation. The other group (second) was infested twice with larvae, first on the left flank 4 weeks after the BMMC transplantation, and 2 weeks later, re-infected with larvae on the BMMC-injected site of the right flank. The relative tick repletion in each group is shown, in that the value in the first infestation was defined as 100% (mean ± SEM, *n* = 3–4). **(D)** *Mcpt8^DTR^* mice were infested once (first) or twice (second) with tick larvae. To deplete basophils during the second infestation, they were treated with intravenous injection of diphtheria toxin (DT) 1 day before the second infestation. To reconstitute basophils, basophils isolated from tick-infested WT or HDC KO mice were adoptively transferred to the DT-treated mice 2 h before the second infestation. The relative tick repletion in each experimental group is shown (mean ± SEM, *n* = 3), in that the value in the first infestation of untreated mice was defined as 100%. All the data shown are representative of at least three independent experiments. Statistics: *t-*test in **(A–C)** and one-way ANOVA in **(D)**. N.S., not significant; *p < 0.05; **p < 0.01; ***p < 0.001.

Both mast cells and basophils are crucial for the manifestation of ATR (9). Considering the fact that histamine is also essential for it (Figures 1 and 2), it is likely that histamine derived from either mast cells or basophils or both contributes to ATR. We found that BMMCs and BMBAs produced similar levels of histamine when stimulated with IgE and corresponding antigens (Figure 3A). Thus, both cell types can function as the provider of histamine necessary for the manifestation of ATR. As expected, both BMMCs and BMBAs derived from HDC-deficient mice failed to produce histamine (Figure 3A).

We first examined the contribution of mast cell-derived histamine by means of adoptive transfer of HDC-sufficient or deficient mast cells to mast cell-deficient mice. As reported previously (9), mast cell-deficient *Kit*^W-sh/W-sh^ mice failed to manifest ATR in the second infestation (Figure 3B). Adoptive transfer of BMMCs derived from WT mice conferred ATR on mast cell-deficient mice, as expected (Figure 3C). Intriguingly, this was also true for the adoptive transfer of BMMCs derived from HDC-deficient mice (Figure 3C), indicating that mast cell-derived histamine was dispensable for the manifestation of ATR.

We next examined the role of basophil-derived histamine in ATR (Figure 3D). We previously demonstrated that the first tick infestation induces the generation and distribution of tick antigen-specific CD4^+^-resident memory T cells in the skin throughout the body and that these memory cells are necessary for the recruitment of basophils to the second tick feeding site (19). Moreover, because basophils have very short life span (~60 h) in contrast to mast cells (21), we cannot apply the mast cell transfer protocol to basophils. To overcome these obstacles, we took advantage of *Mcpt8*^DTR^ mice in that only basophils express human DT receptor and, therefore, can be depleted by DT treatment of mice (9). *Mcpt8*^DTR^ mice were first infested with tick larvae, and then treated with DT 1 day before the second infestation to deplete basophils and leave CD4^+^-resident memory T cells intact in the skin. Just before the second infestation, basophils freshly isolated from WT or HDC-deficient mice that had experienced tick infestation were adoptively transferred to the DT-treated *Mcpt8*^DTR^ mice to reconstitute basophils. As expected, the transfer of WT basophils conferred ATR on the recipient mice (Figure 3D). In contrast, the transfer of HDC-deficient basophils failed to do so (Figure 3D), demonstrating the essential role of basophil-derived histamine in the manifestation of ATR.

### Basophils Accumulate in Larger Numbers and at a Position Closer to Tick Mouthparts Than Do Mast Cells During the second Tick Infestation

**Figure 4:**
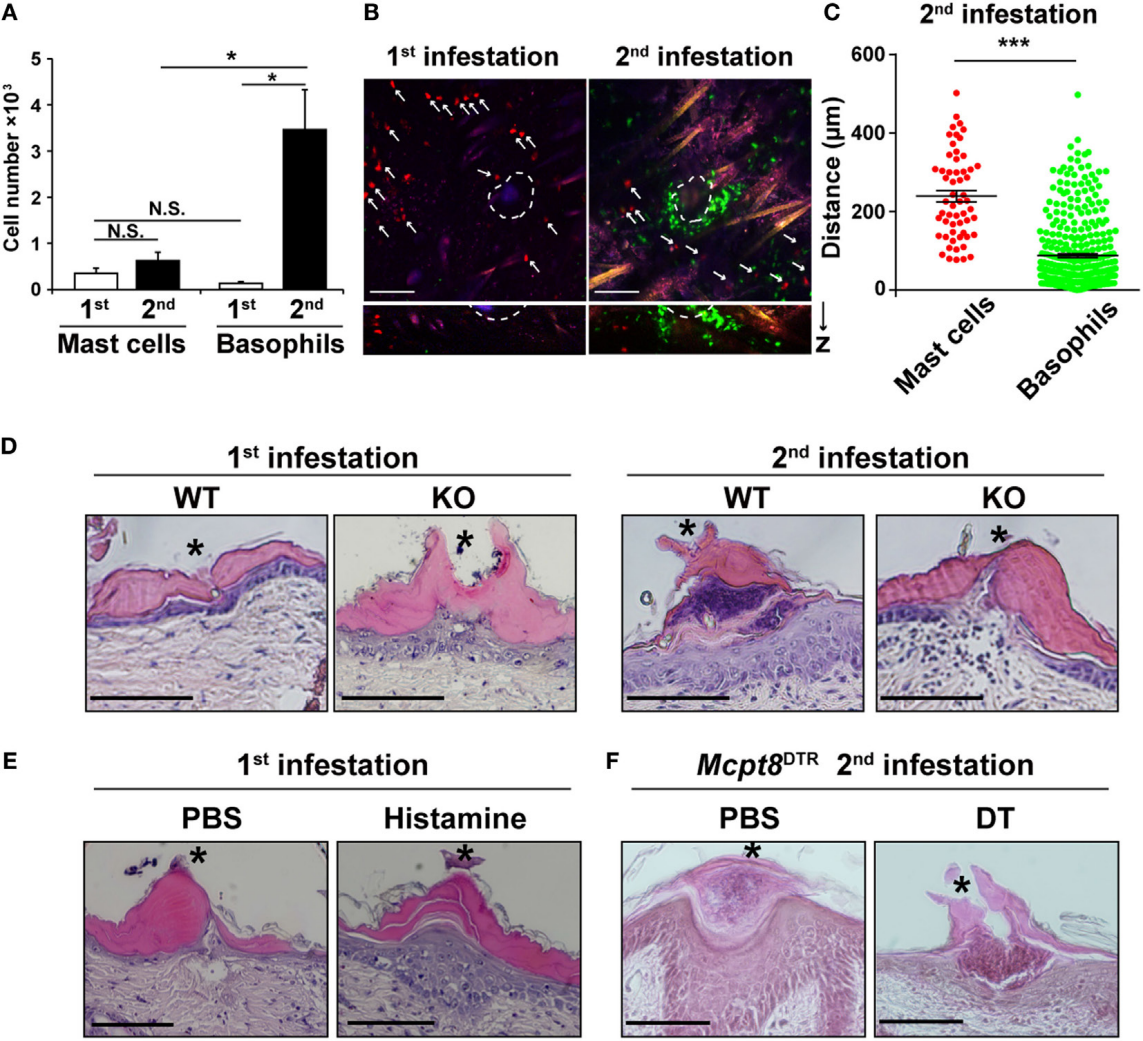
Basophils accumulate in larger numbers and at a position closer to tick mouthparts than do mast cells during the second tick infestation. **(A)** WT mice were infested once or twice with tick larvae. The numbers of basophils and mast cells (mean ± SEM, *n* = 4 each) accumulating at the second tick-feeding site on day 2 of the first or second infestation were examined by using flow cytometry. **(B)** Bone marrow-derived mast cell prepared from CAG-tdTomato transgenic mice were intradermally transferred into the right flank of *Kit^W-sh/W-sh^Mcpt8*^GFP^ mice. Recipient mice were infested once or twice with tick larvae as in Figure 3C and subjected to intravital imaging analysis of basophils (in green) and mast cells (in red, marked with white arrows) at tick-feeding sites on day 2 of the first or the second infestation. Upper pictures show the compilation of 12 horizontal images acquired from 0 to 100 μm deep under the skin. Lower panels show vertical images of upper pictures. Dashed circular lines indicate sites where tick mouthparts were inserted into the skin. Scale bars indicate 100 μm. **(C)** The distance of each basophil and mast cell away from the nearest tick mouthpart was measured in the images of the second tick-feeding site (mean ± SEM, 58 mast cells and 371 basophils were analyzed). **(D)** Skin sections of tick feeding sites in WT and histidine decarboxylase KO mice on day 2 of the first or second infestation were stained with hematoxylin and eosin. Asterisks indicate sites where tick mouthparts were inserted into the skin. Scale bars indicate 200 µm. **(E)** Histamine or control PBS was injected intradermally beneath the first tick-infested site once a day for 3 days, starting 1 day before the infestation. On day 2 of the first infestation, skin sections of tick feeding sites were stained with hematoxylin and eosin as in **(D)**. **(F)** *Mcpt8^DTR^* mice were treated with diphtheria toxin or control PBS 1 day before the second infestation. Skin sections of tick feeding sites were stained with hematoxylin and eosin as in **(D)**. All the data shown in **(A–F)** are representative of at least three independent experiments. N.S., not significant; *p < 0.05; ***p < 0.001.

We sought to find the reason why histamine derived from basophils rather than mast cells plays an important role in ATR (Figures 3C,D), in spite of the fact that both cell types can produce histamine at comparable levels *in vitro* (Figure 3A). Flow cytometric analysis of cells present at the tick feeding sites revealed that the number of mast cells did not significantly increase at the second tick feeding site, compared to that at the first site (Figure 4A). Basophils were hardly detected at the first tick feeding site, but they accumulated five times as many as mast cells at the second tick feeding site (Figure 4A).

We then examined the localization of mast cells and basophils at the tick feeding site by using confocal fluorescence microscopy. To visualize and distinguish them, we generated mice in that mast cells express tdTomato (in red) while basophils express GFP (in green). In accordance with the flow cytometric analysis (Figure 4A), basophils were barely detected at the first feeding site while they accumulated at the second feeding site and made a cluster, surrounding a tick mouthpart, within the epidermis (Figures 4B,D). In contrast, mast cells were mostly scattered and present in the dermis rather than epidermis regardless of the first and second infestation (Figure 4B, not all data shown). On average, mast cells were localized more distantly from the tick mouthpart, compared to basophils (Figures 4B,C). Taken together, basophils accumulated at the second tick feeding site in larger numbers and more closely to the tick mouthpart than did mast cells. This may explain the predominant role of basophils in the manifestation of ATR through histamine release.

Histopathological examination of tick feeding sites in WT mice revealed that the epidermis was much thicker at the second tick feeding site than the first one (Figure 4D). Of note, such thickening of the epidermis was not observed at the second tick feeding site of HDC-deficient mice (Figure 4D), suggesting the possible contribution of histamine to epidermal hyperplasia. Indeed, repeated intradermal injection of histamine, but not control PBS, beneath the tick-infested site before and during the first infestation in WT mice resulted in epidermal hyperplasia (Figure 4E), in parallel with the manifestation of tick resistance (Figure 1B). Moreover, DT-mediated basophil depletion before the second infestation in *Mcpt8*^DTR^ mice abolished the development of epidermal hyperplasia (Figure 4F). Taken together with the observation that histamine derived from basophils rather than mast cells was required for ATR (Figures 3C,D), one may assume that basophil-derived histamine plays an important role in ATR, perhaps through the induction of epidermal hyperplasia that might interfere with tick attachment or blood-feeding in the skin.

## Discussion

A previous study demonstrated the prerequisite role of both tick antigen-specific IgE and mast cells in the manifestation of ATR in mice (8). Considering the fact that the tick resistance is reportedly correlated with the amounts of histamine at the tick feeding site in cattle and guinea pigs (12, 15), it was hypothesized that histamine released from mast cells stimulated with IgE and tick antigens contributes to ATR in mice. In contrast, in guinea pigs, the important role for basophils in the manifestation of ATR was demonstrated (7). We have recently established a basophil-specific mAb and genetically engineered mice deficient for only basophils, which enabled us to detect the accumulation of basophils at the second tick feeding site in mice and identify a crucial role for basophils, in addition to mast cells, in ATR (9). However, it remained uncertain how basophils and mast cells contribute to ATR. In the present study, we demonstrated that histamine crucially contributes to ATR in mice, in accordance with previous reports in cattle and guinea pigs. Importantly, skin-infiltrating basophils rather than tissue-resident mast cells turned out to be the major source of histamine responsible for ATR, in contrast to the case of local and systemic anaphylaxis where mast cell-derived histamine is the major player.

Numerous degranulated mast cells were detected at the tick feeding sites of resistant cattle and mice (8, 22). Transfer of sera from previously infested mice but not heat-treated sera conferred ATR and the degranulation of mast cells at the feeding site on naive recipient mice (8), suggesting IgE-mediated activation and degranulation of mast cells at the tick feeding site, likely leading to histamine release from mast cells. Nevertheless, the present study demonstrated little or no contribution of mast cell-derived histamine to ATR, in contrast to basophil-derived one. This appears to be consistent with our previous finding that IgE-mediated activation of basophils but not mast cells is pivotal for the manifestation of ATR even though the presence of mast cells is necessary for it (9). In the present study, we compared basophils and mast cells in terms of their numbers and localization at the second tick feeding site to understand the reason why histamine released from basophils rather than mast cells plays a predominant role in ATR. Compared to mast cells scattered in the dermis, approximately five times more basophils accumulated in the epidermis of the second tick feeding site and made a cluster, surrounding a tick mouthpart (Figures 4A,B). Given that histamine has a very short half-life, the action of histamine is thought to be effective only within a short distance away from activated basophils and mast cells. Even though the exact target of histamine remains to be investigated, the accumulation of higher number of basophils and their localization closer to the tick-feeding spot, in comparison to mast cells, appear to make basophil-derived histamine much more effective than mast cell-derived one. The role of mast cells in ATR remains to be investigated. We previously reported that the number of basophils accumulating at the tick-feeding site during the second infestation is comparable between mast cell-sufficient and -deficient mice (9). On closer examination with confocal fluorescent microscopy, basophils accumulating at the tick-feeding site in mast cell-deficient mice appeared to be more motile and less-clustered around a tick mouthpart compared to those in mast cell-sufficient mice (data not shown). This suggested that mast cells might contribute to ATR by regulating basophil behavior directly or indirectly, even though the exact mechanism remains to be clarified.

Tick saliva contains the lipocalin family proteins characterized by histamine-binding activity that inhibits the effects of histamine (23), implying that histamine produced by host cells plays an important role in the defense against tick feeding. Previous studies suggested that histamine induces itching in the skin and, therefore, host grooming response, leading to removal of ticks (24). Under our experimental conditions, ticks were placed inside of an acryl ring attached to the skin and, therefore, the effect of host grooming on tick feeding was minimized, suggesting other mechanisms underlying histamine-mediated ATR. An *in vitro* study using a membrane blood-feeding model with electrophysiological recording of tick feeding demonstrated that histamine directly causes diminished blood uptake and salivation by *Dermacentor andersoni* ticks (25). This may not be the case in the present study because mice deficient for H1R failed to manifest ATR (data not show), suggesting that histamine acts on host cells *via* H1 receptor, not directly on ticks. In cattle, repeated injection of histamine beneath tick attachment sites promotes early detachment of *Boophilus microplus* larvae without any apparent effect on the increase of their body weight (14). Such detachment of ticks was observed when cattle were treated with histamine within 24 h but not 48 h after the initiation of tick infestation, suggesting the effect of histamine at the attachment but not blood-feeding phase. We also observed the similar effect of histamine injection on the tick attachment but not blood feeding in mice (data not shown).

In the present study, we detected the thickening of the epidermis at the second tick feeding site and the formation of basophil cluster within the thickened epidermis, as reported previously in guinea pigs (10). Notably, mice deficient for either HDC or basophils failed to form such thickened epidermis. Moreover, repeated injection of histamine, but not control PBS, at the first tick feeding site in WT mice resulted in thickened epidermis. Considering previous reports that murine keratinocytes express functional H1 receptors (26) and that histamine stimulates the proliferation of murine epidermal keratinocytes (27, 28), histamine released from basophil clusters in the epidermis likely promotes the thickening of the epidermis at the second tick feeding site. It has been demonstrated that *H. longicornis, B. microplus*, and *D. andersoni* larval ticks are highly responsive to histamine in the induction of tick resistance while *Ixodes holocyclus* and *Amblyomma americanum* ticks are less reactive to histamine (14, 15, 29, 30). The former species have shorter mouthparts than the latter (31), suggesting that the thickening of the epidermis induced by histamine can prevent the former’s but not the latter’s mouthparts from penetrating into the dermis through the epidermis to form blood pools. This may account for the different responsiveness to histamine among tick species in the induction of ATR.

In conclusion, we explored possible effector molecules involved in ATR in mice and demonstrated that histamine released from basophils rather than mast cells plays a crucial role in it. Further studies on the detailed mechanism underlying histamine-induced tick resistance may help develop therapeutics to prevent tick infestation and tick-borne diseases.

## Ethics Statement

This study was carried out in accordance with the guidelines of the Tokyo Medical and Dental University for animal care. The protocol was approved by the Institution Animal Care and Use Committee of Tokyo Medical and Dental University.

## Author Contributions

YT, TO, SY, YK, KM, YY, HO, TA, HK, NW, and HaK designed the research. YT, TO, SY, and ER performed experiments and analyzed data. KY and KI prepared ticks suitable for infestation. HaK and SY supervised the work, and wrote the manuscript. All authors provided critical review of the manuscript.

## Conflict of Interest Statement

The authors declare that the research was conducted in the absence of any commercial or financial relationships that could be construed as a potential conflict of interest.

## Abbreviations

ATR, acquired tick resistance; IgE, immunoglobulin E; HDC, histidine decarboxylase; DT, diphtheria toxin; BMMC, bone marrow-derived mast cell; BMBA, bone marrow-derived basophil.
