# Supplementary figures and images for: Histamine Released From Skin-Infiltrating Basophils but Not Mast Cells Is Crucial for Acquired Tick Resistance in Mice
Source: Front Immunol. 2018 Jul 3;9:1540. doi: 10.3389/fimmu.2018.01540 (PMC6043789; doi:10.3389/fimmu.2018.01540)

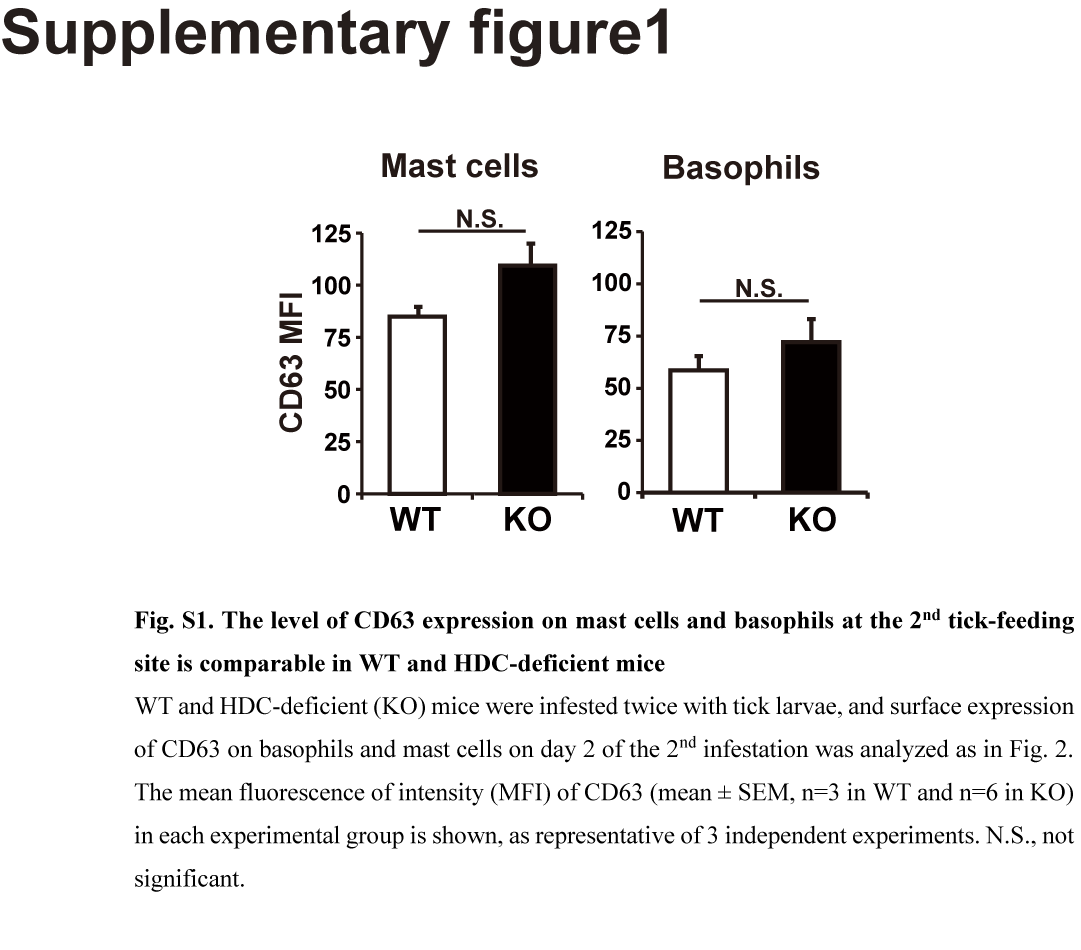

Supplement: Supplementary file 1 [file data_sheet_1.tif]
